# Salivary microbiota profile in adult and children population according to active dentin caries: a metagenomic preliminary analysis

**DOI:** 10.3389/froh.2025.1599925

**Published:** 2025-07-28

**Authors:** Nuria Tamayo-Estebaranz, Carolina Muñoz-González, Ana María Gil-Valcárcel, Paula Calvo López-Dávalos, Andrea Martín-Vacas, Marta M. Paz-Cortés, Juan Manuel Aragoneses

**Affiliations:** ^1^ Programa de Doctorado en Ciencias de la Salud, Departamento de Medicina y Especialidades Médicas, Facultad de Medicina y Ciencias de la Salud, Universidad de Alcalá, Alcalá de Henares, Spain; ^2^ Facultad de Odontología, Universidad Alfonso X El Sabio, Villanueva de la Cañada, Spain; ^3^ Instituto de Investigación en Ciencias de la Alimentación, CSIC-UAM, Madrid, Spain; ^4^ Department of Dental Research, Federico Henriquez y Carvajal University, Santo Domingo Oeste, Dominican Republic

**Keywords:** dentistry, microbiology, caries, oral pathology, periodontitis, metagenomic, saliva

## Abstract

**Objectives:**

The aim of this study was to investigate the relationship between active dentin caries (ADC), salivary biochemical parameters, and salivary microbiota composition in Spanish children and adults.

**Methods:**

Saliva samples were collected from 80 subjects (40 adults and 40 children) divided between ADC and non-ADC. Salivary biochemical determination was performed by analysing total protein content (TPC) and total antioxidant activity (TAC) in saliva supernatants. DNA was obtained from the pellet of saliva samples using the Bacterial DNA kit and analysed with the Illumina NextSeq platform from all participants. Alpha diversity (Chao, Observed Features, Shannon and Simpson indices) and beta diversity (PCoA plot and PERMANOVA procedure) were analysed. In addition, Linear Discriminant Analysis Effect Size (LEfSe) was used to identify differential taxa between groups. All statistical analysis were performed with a 95% confidence level (*p* < 0.05).

**Results:**

No significant associations were found between ADC and salivary biochemical markers in either the adult or pediatric age group, suggesting that these parameters alone may not sufficiently reflect cariogenic activity. Microbiota analysis at the phylum level did not show significant correlations with ADC; however, distinct associations appeared at the genus and species levels. In adults, several genera (*Corynebacterium*, *Porphyromonas*, *Tannerella*, *Catonella*, *Filifactor*, *Parvimonas*, and *Dialister*) were positively associated with ADC, reflecting a shift towards a dysbiotic microbiome composition that overlaps with periodontal and endodontic pathologies. Conversely, *Haemophilus* was negatively correlated with ADC, potentially indicating a protective role. At the species level, a positive correlation with ADC was found with *Porphyromonas gingivalis*, *Porphyromonas endodontalis, Peptostreptococcus stomatis*, *Leptotrichia buccalis*, *Prevotella oris*, or *Corynebacterium matruchotii* in the adult population. In children, microbial associations with caries were more limited, with *Scardovia*, a well-known acidogenic genus, positively correlated with ADC, and *P. stomatis* showing a negative association. Interestingly, *P. stomatis* exhibited opposite correlations in adults and children, possibly reflecting age-specific ecological roles. No significant differences in alpha or beta diversity were found either in adults or children participants.

**Conclusions:**

Overall, these findings highlight a stronger and more diverse association between salivary microbiota and caries in adults compared to children. These results underscore the importance of age-specific microbial signatures in the aetiology of dental caries. The obtained differences suggest that caries development in adults may involve broader dysbiosis involving proteolytic and anaerobic organisms in addition to acidogenic species.

## 1 Introduction

The oral cavity is one of the most important habitats of the human body and one of the richest environments in microorganisms, consisting of bacteria, fungi, viruses, and archaea (1–3). It is formed by an ecological community of commensal, symbiotic, and pathogenic microorganisms (4) with a significant impact on both oral and general health and is a potential diagnostic indicator for various diseases such as cardiovascular disease, rheumatoid arthritis, cancer, or diabetes, among others (5–7). The oral environment constantly transforms with age, and, consequently, the microbiota also changes (8). At birth, most children do not have a colonized microbiome, and during the first two months of life, bacteria colonize the mucosal surfaces, and later, with the eruption of primary teeth, these surfaces are also colonized (8). During childhood, primary dentition exfoliates, leading to mixed dentition and finally to permanent dentition (2), with observed changes in bacterial colonization at different dentition stages (6, 9), resulting in known differences between the microbiota of children and adults (10, 11). In this regard, Ling et al. (12) observed that *Actinobacteria* (syn. *Actinomycetota*), *Bacteroidetes* (syn. *Bacteroidota*), and *Fusobacteria* (syn. *Fusobacteriota*) were overrepresented in children, whereas *Firmicutes* (syn. *Bacillota*) and *Proteobacteria* (syn. *Pseudomonadota*) were more abundant in adults.

Although oral microbiota is resistant to minor ecological changes, factors such as diet, environment, inadequate oral hygiene (13), the use of medications or prolonged use of antibiotics (8) can also induce structural changes in the microbiota (14) leading to dysbiosis of the resident microbiota (4), which can result in caries (1).

Untreated dental caries is the most prevalent condition worldwide and is estimated to affect 2.5 billion people of which 530 million are children (15). Although *Streptoccocus* is the main genera related to dental caries, other microorganisms have been identified in relation to the presence of caries lesions (i.e., *Fusobacterium, Prevotella, Leptotrichia, Veillonella, Bifidobacterium,* and *Capnocytophaga* species) (4). However, several important limitations in the existing studies on the oral microbiota and its association with dental caries have to be considered. Many of these investigations rely on non-omics-based techniques, which restrict the detection and characterization of non-culturable bacteria, thus providing an incomplete picture of the microbial diversity involved. Furthermore, most studies have been conducted in Asian populations, with limited data available from Spanish or broader European cohorts, making it difficult to generalize findings across different populations. In addition, there is a lack of studies that simultaneously analyse both children and adults, despite known age-related differences in oral microbiota composition. In this sense, confounding factors such as sex and age are often not adequately controlled or considered in the analysis, potentially biasing the results and limiting the reliability of observed associations.

Another aspect to consider is the site of sample collection. In this regard, saliva is a biological fluid that bathes the oral cavity, and it is essential for maintaining both oral and general health (14). It is estimated that over 700 bacterial species coexist in the oral cavity (16), among them, only two-thirds belong to *in vitro* cultivable species (17). The rich microbiota provides a composite representation of microorganisms from diverse niches within the oral cavity (18) and reflects local alterations of the supragingival and subgingival microbiota (4). Salivary collection is non-invasive, simple, and painless collection method, which makes it particularly suitable for studies involving children (16). Moreover, it has been reported that saliva composition parameters, such as total protein content and antioxidant capacity, are associated with the composition of the salivary microbiota (19) which may in turn influence susceptibility to oral conditions such as caries. These biochemical properties could potentially modulate the oral environment and shape microbial communities, highlighting the importance of integrating salivary biomarkers into microbiome research. These gaps underscore the need for more comprehensive, omics-based, and demographically diverse research that considers developmental stages, relevant confounding variables, and optimized sampling strategies to better understand the complex interactions between the oral microbiome and dental caries.

This study aimed to investigate the relationship between salivary microbiota composition, based on 16S rRNA gene V3–V4 amplicon sequencing, and the presence of active dentin caries (ADC), in Spanish children and adults. Additionally, the relation between salivary parameters, including pH, flow rate, total protein content, and antioxidant activity, and caries status was evaluated in both age groups.

## 2 Methods

A case-control, cross-sectional, observational, and analytical study was conducted, with two parallel and matched arms. The study design was approved by the Bioethics Committee of the San Carlos Clinical Hospital (code 22/334-E, 31st May 2022) complying with current Spanish and European regulations on personal data protection and the principles of the Declaration of Helsinki for research involving human subjects. Additionally, the STROBE guidelines for cross-sectional studies were followed as reporting guidelines (20) (Supplementary Table S1).

### 2.1 Study population

The subjects in this study were adults (*n* = 40) and children (*n* = 40) attending dental check-ups at the dental clinic of Alfonso X El Sabio University (UAX) in Madrid, Spain. Subjects aged 6–12 years were included for the paediatric population and 20–40 years for the adult population, who voluntarily agreed to participate in the study. Additionally, the main groups were subdivided into non ADC (non-ADC) and ADC categories based on their active caries index. According to the International Caries Detection and Assessment System (ICDAS), caries presence was classified in initial or enamel caries (healthy tooth according to OMS code) as ICDAS codes 1–3, or ADC as ICDAS Codes 4–6 (21). The non-ADC consisted of volunteers with non caries (ICDAS codes 0–3), while the ADC group included volunteers with active caries in dentin (ICDAS codes 4–6). This resulted in four groups of volunteers: children with non-ADC (*n* = 20), children with ADC (*n* = 20), healthy adults with non-ADC (*n* = 20), and adults with ADC (*n* = 20). All groups are balanced in terms of sex. In addition, the Community Periodontal Index (CPI) were calculated to assess oral health status of the volunteers (22, 23). Subjects with behavioural problems, smokers, systemic diseases, pregnant or during lactation, chronic medication or those who had taken antibiotics in the last three months were excluded.

Previous similar research studied 30–50 children (13, 24–27) or 46 adults (28), respectively. Due to differences in study procedure and outcomes and the preliminar feature of the research, we decided to calculate the needed sample size theoretically, based on caries prevalence. The sample size was calculated assuming an infinite reference population (average patient volume data from the dental clinic not available) and a caries prevalence of 95% (100% in the adult population, 90% in the paediatric population) (29). A random sample of 74 individuals was found sufficient to estimate, with 95% confidence and a precision of ±5 percent points. The anticipated replacement rate is projected to be 1%, to resolve possible sample losses during the collection or sampling procedure. To ensure sample homogeneity, the decision was made to increase the sample size to 80 subjects, evenly divided between adult and paediatric populations, (*n* = 40 in each population group) adjusted by sex. In each population group, the same subject amount of each oral health status was selected, so finally 20 children with non-ADC, 20 children with ADC, 20 adults with non-ADC and 20 adults with ADC were included.

The sampling method was non-probabilistic consecutive cases between November 2022 and January 2023, until reaching the predetermined number. Study subjects over 16 years old were initially informed verbally and given a Patient Information Sheet and an Informed Consent Form, which had to be completed before study participation. In cases where the subjects were under 12 years old, these steps were performed and authorized by their parents or legal guardians. For children aged 12 years, the child's acceptance, as well as that of the parents, was essential for participation in the study.

### 2.2 Health data and salivary collection

Oral and general health data were obtained directly from the study participants through oral survey by the principal investigator (N.T.-E.), including age and sex. Oral health data (caries presence and CPI) were recorded following WHO and ICDAS guidelines (21, 22). Data regarding systemic health, medication intake, smoking habits, alcohol intake and current health status were also collected to appropriately select the sample.

Saliva samples were collected at the dental clinic facilities of UAX by the study investigators between 8 and 11 am, confirming through interviews with the subjects that they maintained fasting and did not brush their teeth for at least 1 h before the collection. Individuals were instructed in the collection of samples by unstimulated saliva expectoration into collection tubes (Labbox, Barcelona, Spain) for 10 min. Immediately after saliva collection, the salivary pH was measured, and salivary flow was calculated by the difference in weight between the empty tube and the tube after spitting out the saliva and expressed as ml/min, assuming 1 g being equal to 1 ml. Immediately, salivary samples were frozen at −20°C. Subsequently, saliva samples were centrifuged (15,000 g for 15 min at 4°C), and the supernatant and pellet were aliquoted separately and stored at −80°C until analysis, to prevent potential changes in their composition and/or properties.

### 2.3 Biochemical analysis of salivary samples

Biochemical determination was performed by analysing total protein content (TPC) and total antioxidant activity (TAC) in saliva supernatants. TPC was measured using the commercial Pierce^TM^ BCA Protein Assay Kit (Pierce ThermoScientific, Rockford, IL, USA). Antioxidant activity (TAC) was measured using the Ferric Reducing Ability of Plasma (FRAP) assay method described by Palomar-Bonet et al. (30).

### 2.4 DNA extraction, sequencing, and data processing

Total DNA was obtained from the pellet of saliva samples using the Bacterial DNA kit (D3350-02, E.Z.N.A™, OMEGA bio-tek, USA), following the manufacturer's instructions. Pellets were resuspended in 100 μl TE buffer containing lysozyme (10 mg/ml), and microbial cells were lysed by mechanical disruption with glass beads (0.1 mm zirconia/silica diameter), using a FastPrep disruptor (QBioGene, Irvine, CA, USA) at a speed of 6 m/s for 30 s. DNA retained on the HiBind DNA column of the kit was eluted twice with 50 μl elution buffer. The concentration and purity of genomic DNA were measured using a NanoDrop™ ND-1000 UV spectrophotometer (Nano-Drop Technologies, Wilmington, DE, USA). DNA samples were analysed via amplicon-based metagenomic sequencing of the 16S rDNA V3–V4 region, performed by Novogen (Cambridge, UK) on an Illumina platform, to generate 250 bp paired-end reads. Bacterial taxonomy was assigned to the obtained ASVs (Amplicon Sequence Variants) by using the QIIME2 software (https://qiime2.org/). ASVs with abundance lower than 0.1% were removed. Subsequent analyses of alpha diversity (calculation of Chao, Observed Features, Shannon and Simpson indices) were performed based on the normalized data.

A beta-diversity analysis was performed, to assess differences in the multivariate microbiota composition between study groups. In order to evaluate the complexity of the community composition and compare the differences between samples, beta diversity was calculated based on weighted and unweighted unifrac distances in QIIME2 software. Cluster analysis was performed with principal component analysis (PCA), which was applied to reduce the dimension of the original variables using the *ade4* package and *ggplot2* package in R software (Version 3.5.3). Principal Coordinate Analysis (PCoA) was performed in the entire group to obtain principal coordinates and visualize differences of samples in complex multi-dimensional data. A matrix of weighted or unweighted unifrac distances among samples obtained previously was transformed into a new set of orthogonal axes, where the maximum variation factor was demonstrated by the first principal coordinate, and the second maximum variation factor was demonstrated by the second principal coordinate, and so on.

### 2.5 Statistical and bioinformatic analyses

Statistical analysis was performed XLSTAT program (version 19.01, Addinsoft, Paris, France), R Software (Version 2.15.3) and IBM® SPSS® Statistics software (version 29.0.2.0, IBM), with a confidence level of 95% (significance set at *p* < 0.05). Adjustment to a normal distribution was analysed with Kolmogorov–Smirnov and Shapiro–Wilk tests. Separate analysis was performed for adults and children. Intragroup differences in biochemical salivary variables were evaluated with Student's T test, as biochemical outcomes followed a normal distribution. Linear Discriminant Analysis Effect Size (LEfSe LDA Effect Size) (version 1.0), with LDA score threshold of 4, was performed for identification of genomic features. Due to non-parametric distribution of evaluated variables, differences between salivary microbiota between study groups (non-ADC and ADC) were analysed using Mann–Whitney test and the behaviour of outcomes was evaluated with Spearman correlation analysis. Regarding the beta-diversity analysis, the three-dimensional PCoA results were displayed using QIIME2 package, while the two-dimensional PCoA results were displayed using *ade4* package and *ggplot2* package in R software (Version 2.15.3). To study the significance of the differences in community structure (beta-diversity) between groups, the *adonis* (PERMANOVA) and *anosim* functions in the QIIME2 software were used to do analysis. To find out the significantly different species at each taxonomic level (Phylum, Class, Order, Family, Genus, Species), the R software (Version 3.5.3) was used to do MetaStat and T-test analysis.

## 3 Results

### 3.1 Sample description

Saliva samples from 40 adults and 40 children were analysed, with each age group evenly divided into ADC (*n* = 20) and non-ADC (*n* = 20) individuals. The groups were balanced by sex, and the age of both study and control samples was homogeneous within the adult (*p* = 0.602) and child (*p* = 0.192) groups (Table 1). No differences in BMI index were found (*p* = 0.102 and *p* = 0.779, respectively for adult and child population). As expected, the ADC and non-ADC groups significantly differed in the number of active caries in both children and adults (*p* < 0.001 for both groups). In this regard, the ADC group presented significantly higher values of dentin caries and total caries than the non-ADC group, both in adult and children population Mean CPI was found higher in ADC than non-ADC adult group (*p* = 0.007) but no differences were found among children (*p* = 0.429).

**Table 1:** Demographic characteristics, caries and periodontal status of the study participants.

	Adults			Children		
	ADC Mean ± SD	Non-ADC Mean ± SD	*p* value*	ADC Mean ± SD	Non-ADC Mean ± SD	*p* value
Sex						
*N* (F)	10	10	–	10	10	–
*N* (M)	10	10	–	10	10	–
Age	31.00 ± 6.32	30.15 ± 5.60	0.602	7.85 ± 1.18	8.4 ± 1.23	0.192
BMI index	25.97 ± 3.10	23.95 ± 3.15	0.102	17.37 ± 3.61	16.97 ± 2.22	0.779
Caries						
Dentin	5.25 ± 1.80	0.00 ± 0.00	<0.001*	6.2 ± 3.11	0.0 ± 0.00	<0.001*
Enamel	0.10 ± 0.31	0.05 ± 0.22	0.799	0.45 ± 0.76	0.35 ± 0.49	1
Total^a^	5.35 ± 1.84	0.05 ± 0.22	<0.001*	6.65 ± 3.22	0.35 ± 0.49	<0.001*
CPI	1.75 ± 1.62	0.30 ± 0.66	0.007*	0.2 ± 0.41	0.05 ± 0.22	0.429

SD, standard deviation; F, female; M, male.

Significance values of U Mann–Whitney test between study groups.

^a^ Total. Total caries presence, sum of enamel and dentin caries.

*Significative values *p* < 0.05.

A general visualization of the data obtained was performed, and it was observed that the samples corresponding to four individuals (three adults and one child) showed unmistakable evidence of contamination during collection, extraction, or analysis. Therefore, these samples were not considered during the statistical analysis of the metagenomic data (Supplementary Figure S1).

Regarding salivary parameters (Table 2), salivary flow was 0.6 ± 0.3 ml/min in adults and 0.6 ± 0.4 ml/min in children. These values fall within the normal unstimulated salivary flow range (typically 0.3–0.7 ml/min), indicating no apparent salivary hypofunction in either group. The salivary pH was 7.0 ± 0.3 in adults and 7.1 ± 0.3 in children, also within the expected physiological range (pH 6.2–7.6), suggesting normal acid–base balance in oral fluid. For TPC, values were 1,300.2 ± 456.7 µg/µl in adults and 1,156.3 ± 415.2 µg/µl in children. Although variability exists across individuals and collection methods, both values are within the reported reference ranges for TPC (typically between 500 and 2,000 µg/µl), indicating normal protein composition. Similarly, TAC was higher in adults (367.3 ± 138.6 µM FeSO₄) than in children (281.7 ± 136.8 µM FeSO₄). These values are consistent with ranges reported in healthy populations, although TAC may vary depending on diet, age, and oxidative stress levels. All these parameters showed no statistically significant differences in pH, salivary flow rate, TAC and TPC among the ADC level both in adult and children population (Supplementary Table S2) (*p* > 0.05 for all comparisons).

**Table 2:** Values of the different salivary parameters measured in this study (salivary flow rate, pH, TPC, TAC) in adults and children participants.

	Adults Median ± SD	Children Median ± SD
Flow rate (ml/min)	0.6 ± 0.3	0.6 ± 0.4
pH	7.0 ± 0.3	7.1 ± 0.3
TPC (µg/µl)	1,300.2 ± 456.7	1,156.3 ± 415.2
TAC (µM FeSO4)	367.3 ± 138.6	281.7 ± 136.8

TPC, total protein content; TAC, total antioxidant capacity.

**Figure 1:**
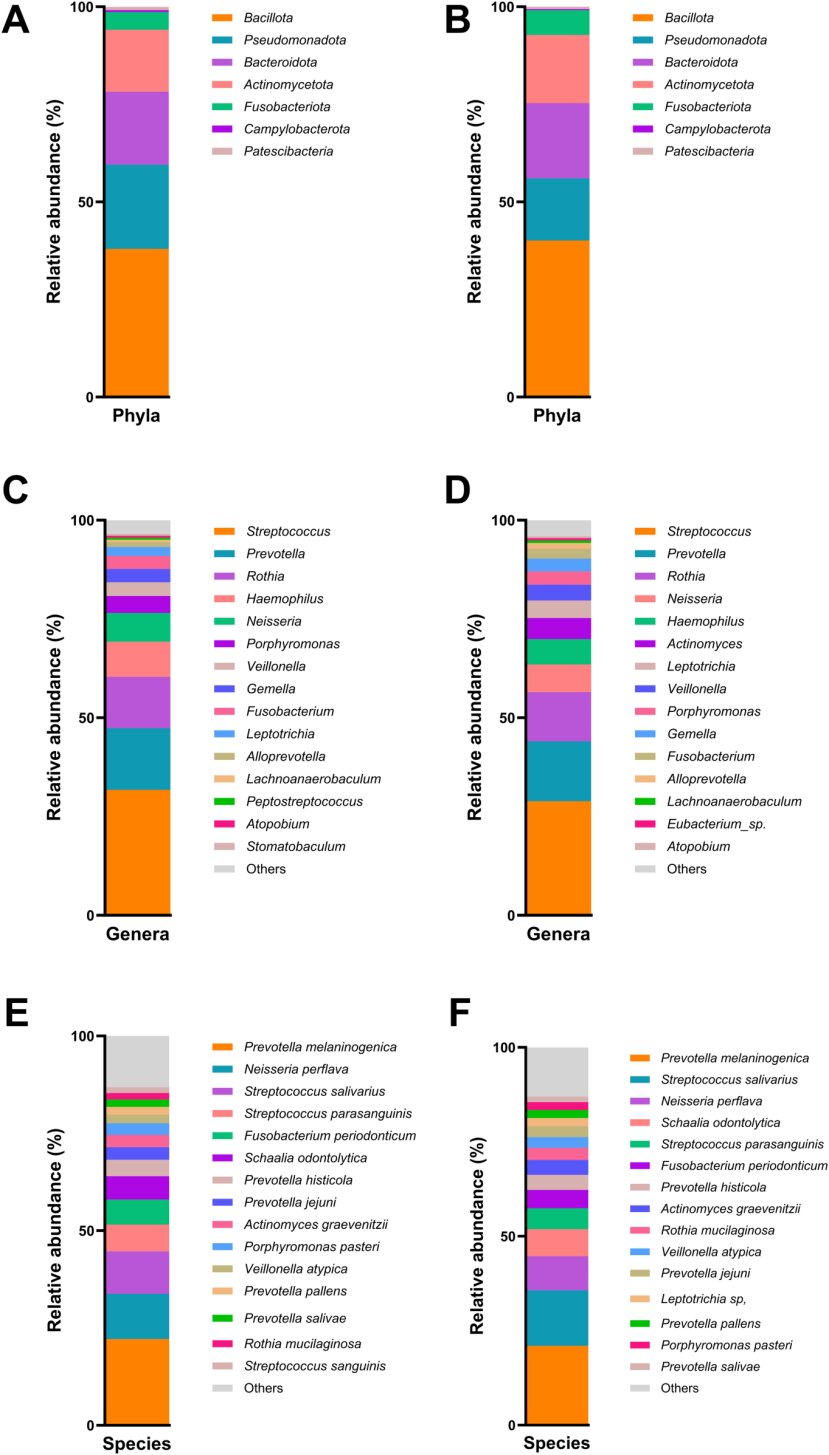
Relative abundance (%) of bacteria phyla in adult group **(A)**, bacteria phyla in children group **(B)**, bacteria genera in adult group **(C)**, bacteria genera in children group **(D)**, bacteria species in adult group **(E)**, and bacteria species in children group **(F)**.

**Figure 2:**
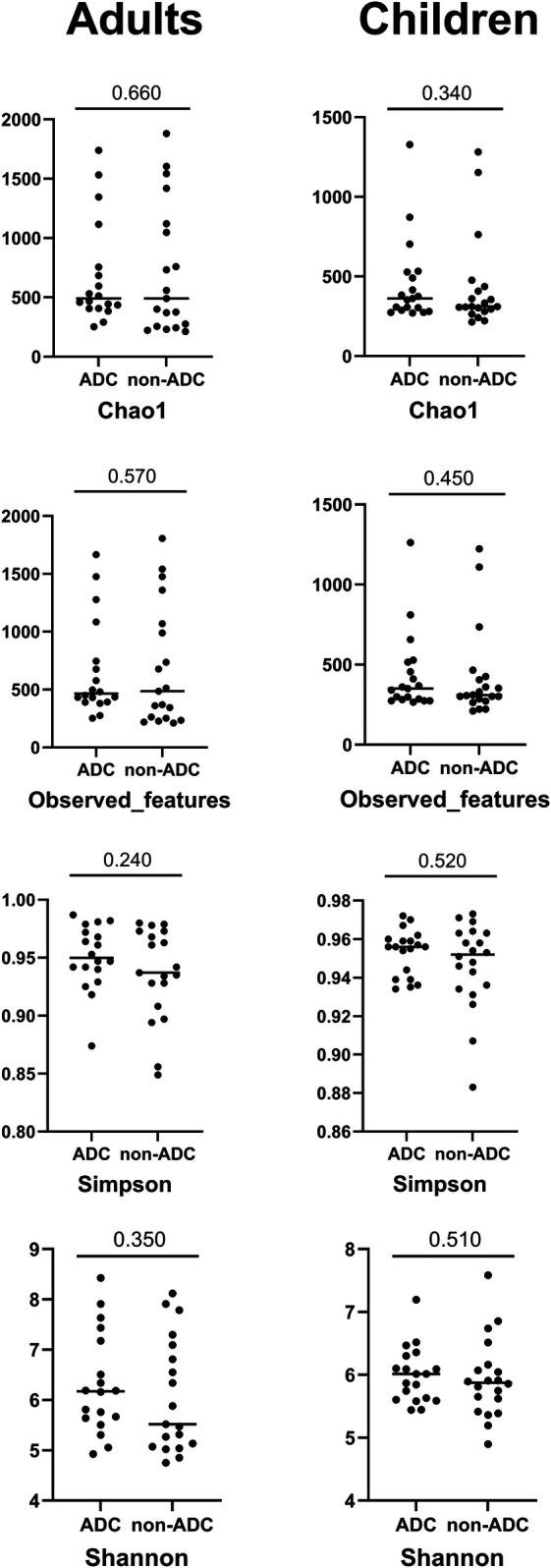
Alpha diversity indices between study groups (ADC and non-ADC) divided according to age categories, and significance values for Mann–Whitney test. No statistically significant differences were found between ADC and non-ADC groups either in adults or children participants in the alpha diversity indices studied.

**Figure 3:**
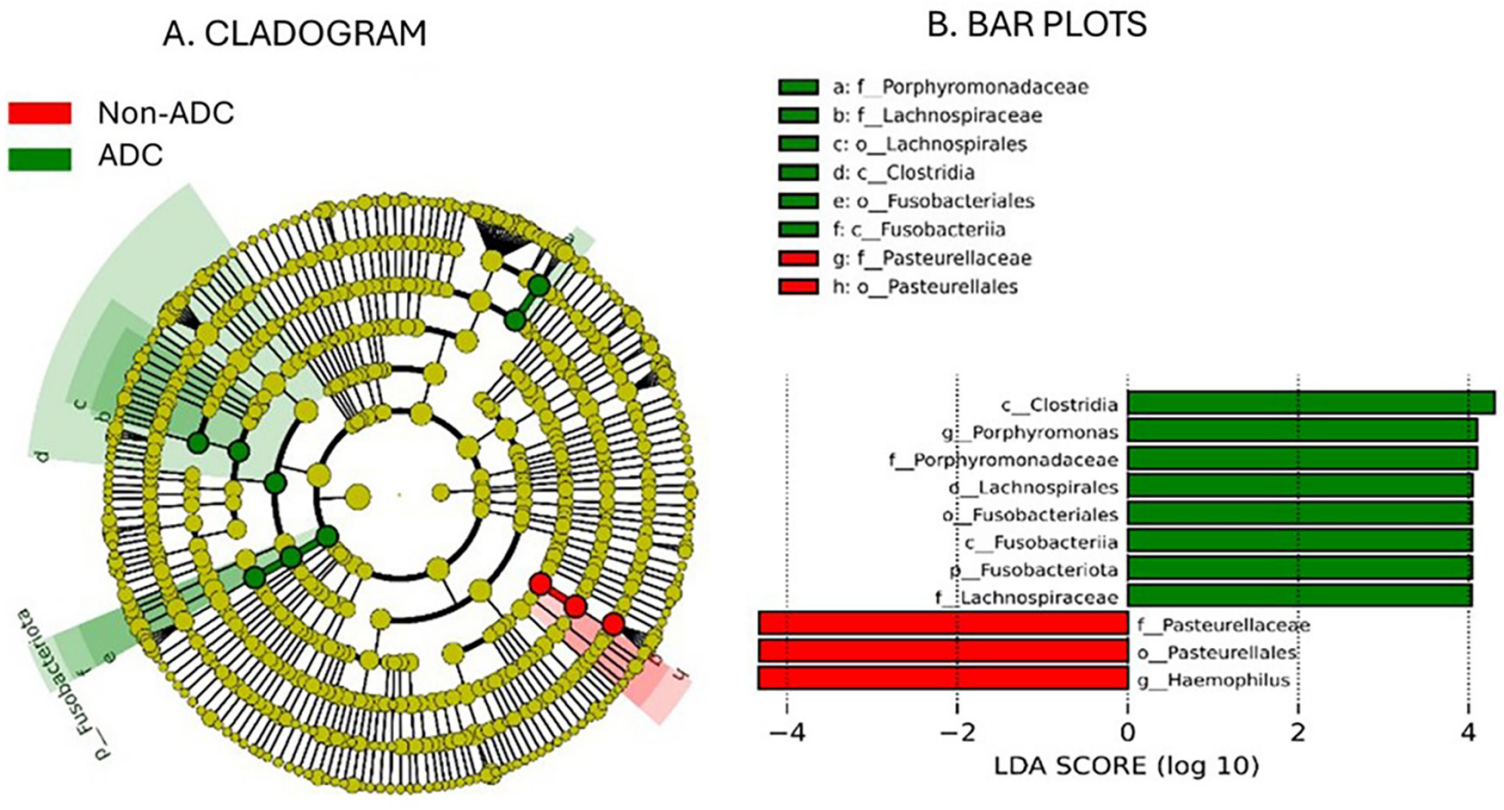
Cladogram analysis **(A)** of 16S rDNA sequences from salivary microbiota in adult group and histogram with bar plots **(B)** showing the significant differences in taxonomy between groups (*p* value < 0.05; LDA score 4.0).

The results of the taxonomic profiling based on saliva samples are summarized in Figure 1. Microbial composition was examined at the phylum, genus, and species levels in both adult and child groups. To ensure clarity and comparability, only taxa with a relative abundance greater than 0.1% in the initial dataset were retained for visualization. Within this filtered subset, relative abundances were recalculated to sum to 100%. At the phylum level (Figure 1A), *Firmicutes* (syn. *Bacillota*) was the most dominant phylum in the adult group, followed by *Proteobacteria* (syn. *Pseudomonadota*) and *Bacteroidetes* (syn. *Bacteroidota*). A similar taxonomic profile was observed in children (Figure 1B), with these three phyla collectively comprising most of the oral microbial community in both groups. This composition is in accordance with previously reported profiles of the human oral microbiome (31). At the genus level, *Streptococcus* emerged as the most abundant genus in both groups (Figures 1C,D), reflecting its well-established dominance in the oral cavity, particularly in early biofilm formation and carbohydrate metabolism (32, 33). Other abundant genera included *Prevotella, Rothia, Haemophilus*, and *Neisseria*, all of which are considered part of the core oral microbiota and are frequently associated with mucosal surfaces and saliva (34). Intra-group analysis of alpha-diversity was conducted separetely on samples from adults and children according to ADC. No statistical significant differences (*p* > 0.05) were found for alpha diversity indices either for adults or children (Figure 2; Supplementary Table S3), suggesting that ADC did not influence the level of bacterial richness present in saliva samples in either children or adults. Regarding the specific taxa that differ between groups (LEfSe), certain taxa were observed to contribute to the separation of ADC groups within the adult population (Figure 3). The most discriminant taxa in the ADC group belonged to the class *Clostridia*, specifically the order *Lachnospirales* and family *Lachnospiraceae*, and to the phylum *Fusobacteria* (syn. *Fusobacteriota*), specifically the class *Fusobacteriia* and order *Fusobacteriales*. Additionally, the family *Porphyromonadaceae* and genus *Porphyromonas* were more abundant in this group. In contrast, the non-ADC group was associated with higher levels of the order *Pasteurellales*, family *Pasteurellaceae*, and genus *Haemophilus.* Regarding children, LEfSe LDA effect size did not reveal any significant results between ADC and non-ADC groups.

**Figure 4:**
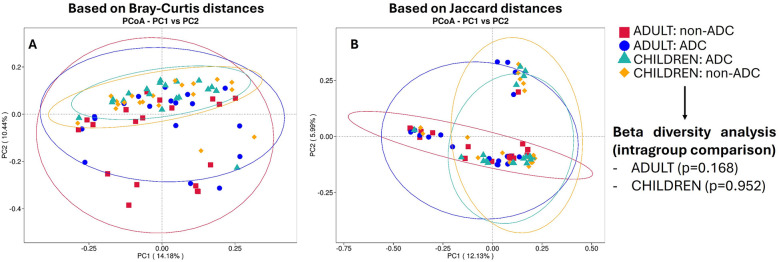
Pcoa plots based on bray-curtis distances **(A)** and on jaccard distances **(B)** No beta diversity differences between age groups were found (PERMANOVA *p* values >0.05). Notably, the most pronounced differences are related to age groups (adults or children), with less distinct clustering based on dental status (ADC or non-ADC).

In order to evaluate the complexity of the community composition and compare the differences between study groups, beta diversity was calculated. No statistically significant differences were found between ADC groups either in the adult (*p* = 0.168) or children (*p* = 0.962) groups studying beta diversity (Supplementary Table S4). A PCoA analysis was conducted to represent the obtained results in the entire group. To visualize the differences in beta diversity between groups, a PCoA based on Bray–Curtis distances (Figure 4A) and another based on Jaccard distances (Figure 4B) were performed. The obtained data indicated that the most pronounced differences were related to age groups, with less distinct clustering based on dental status.

### 3.2 Relationship between salivary parameters and active dentin caries in children and adults

Due to the sample size constraints, correlation analyses were performed to explore potential associations between salivary biochemical parameters and ADC in both adults and children.

**Figure 5:**
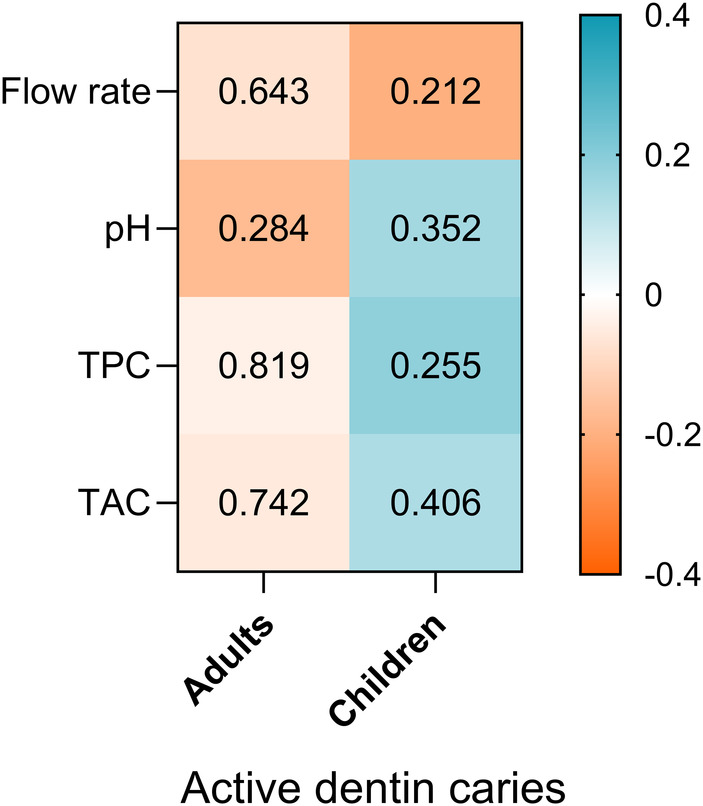
Heatmap of spearman correlation between salivary parameters (flow rate, pH, TPC and TAC) and ADC in adults and children participants. The direction and strength of the correlations are represented by the colour scale, where blue indicates positive correlations and orange indicates negative correlations *P*- values of correlation are shown inside each cell.

As illustrated in Figure 5, no significant correlations were observed between ADC and any of the salivary parameters in either age group (*p* > 0.05 for all comparisons). However, this figure highlights notable differences in the relationship between salivary parameters and ADC in children and adults. In adults, these relationships appear weak, with only a slightly stronger negative correlation observed between salivary pH and the number of active caries, indicating that higher pH levels are associated with fewer carious lesions. In contrast, in children, the associations are somewhat stronger (but not significant) and show a different pattern: positive correlations were observed for pH, total protein content (TPC), and total antioxidant capacity (TAC), whereas flow rate exhibited a negative correlation with ADC.

### 3.3 Relationship between salivary microbiota and active dentin caries, demographic variables, and periodontal index in children and adults

**Figure 6:**
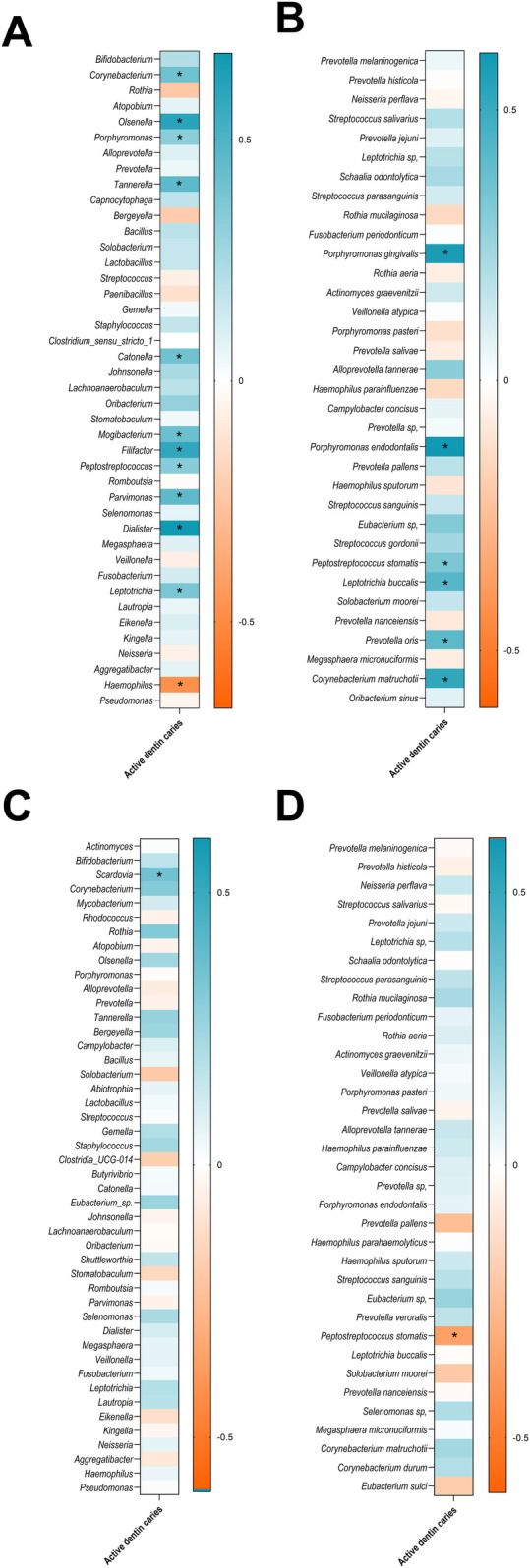
Heatmap of spearman correlations between ADC and the relative abundance of the oral microbiota, showing bacterial genera **(A)**, and species in adults **(B)**,and bacterial genera **(C)**, and species in children **(D)** only taxa with a relative abundance greater than 0.1% are shown. Asterisks indicate statistically significant correlations (*p* < 0.05). The strength and direction of the correlations are represented by the colour scale, with blue indicating positive correlations and orange indicating negative correlations, as shown in the accompanying legend.

Spearman correlation analyses were conducted to investigate potential associations between salivary microbiota composition and ADC in both adults and children (Figure 6; Supplementary Table S5). Neither in adults nor children significant correlations between phylum relative abundance and ADC (*p* > 0.05 for all comparisons). CPI correlation with salivary microbiota composition was also analysed, finding no significant correlation between phylum relative abundance and CPI in children; on the other hand, a significant and negative relationship was found between CPI and *Firmicutes (Syn. Bacillota)* and *Actinobacteriota (Syn. Actinomycetota*)(Supplementary Table S6).

According to the obtained results, notable differences were observed in the association between salivary microbiota composition and the number of ADC in adults compared to children. In adults, a larger number of genera were positively correlated with ADC, including *Corynebacterium*, *Olsenella*, *Porphyromonas*, *Tannerella*, *Catonella*, *Mogibacterium*, *Filifactor*, *Peptostreptococcus*, *Parvimonas*, *Dialister*, and *Leptotrichia* (Figure 6A). Among species, *P. gingivalis*, *P. endodontalis*, *P. stomatis*, *L. buccalis*, *Prevotella oris*, and *C. matruchotii* were positively associated with ADC (Figure 6B). In contrast, *Haemophilus* was the only genus negatively correlated with ADC in adults (Figure 6A). In children, by contrast, the associations between microbiota and ADC were more limited. Only the genus *Scardovia* showed a significant positive correlation with the number of ADC (Figure 6C). At the species level, *P. stomatis* was significantly and negatively associated with ADC (Figure 6D).

Related to periodontal status, in adults many genera were positively correlated with CPI, including *Bifidobacterium, Olsenella, Porphyromonas, Tannerella, Capnocytophaga, Catonella, Johnsonella, Filifactor, Parvimonas, Dialister* and *Leptotrichia*. On the other hand, *Rothia* and *Haemophilus* were negatively correlated with CPI. Among species, *P. gingivalis*, *P. endodontalis, P. stomatis, L. buccalis* and *C. matruchotii* were positively and significantly correlated to CPI. In contrast, in children, the correlations were more limited, with only negative significant correlation between *Porphyromonas* genus, and positive correlation with *Streptococcus salivarius*.

## 4 Discussion

The main objective of this study was to evaluate the relationship between the level of ADC with salivary biochemical parameters and salivary microbiota composition in both, children and adults from Spain. In line with most of the previous work, the present study employed a cross-sectional design, which, although limited in its ability to infer causality, remains a practical and commonly used approach in oral microbiome research. Our sample size (*n* = 80), comprising 40 children and 40 adults evenly distributed by ADC status, is comparable to similar investigations, which have included between 30 and 50 paediatric participants or 40–50 adult subjects. In the present study, we adhered to WHO criteria to define the presence of caries and further classified them into enamel and dentin lesions according to ICDAS, allowing a more precise stratification of disease severity. To minimize confounding variables related to age, participants were selected within a narrow age range, with mean ages of approximately 8 years for children and 30 years for adults. As in many metagenomic studies, 16S rRNA gene sequencing was used to characterize the microbial communities present in saliva. All participants exhibited salivary values within normal ranges for flow rate, pH, TPC, TAC, and microbial composition.

Regarding salivary metagenomic studies, most of them have been conducted in Asian countries such as China (1, 13, 24–26, 28, 35–43) or Korea (44, 45), although they have also been carried out in European countries (9, 10, 12, 14, 16, 17, 27, 46–49), Turkey (50, 51), USA (2, 5, 23, 52, 53) and Australia (54), while only one study conducted in Spain to date (55). It is important to consider the dietary, hygienic and social differences between different races and cultures, which underlines the need to carry out population studies that describe salivary bacterial composition, helping to detect intercultural differences (56). In relation to the design of the published studies, most are cross-sectional, similar to the present study (2, 9, 14, 16, 17, 23–25, 28, 35–43, 46–50, 52, 57), although longitudinal studies have also been conducted (5, 10, 12, 13, 26, 27, 45, 51). Regarding the population characteristics, variables studied, and sample size, we find that the sample size is very heterogeneus, with some studies including only two participants (52), up to studies with 293 subjects (47). In our case, we studied a total of 80 subjects (40 children and 40 adults, evenly divided according to ADC), similar to previous studies, with 30–50 children (13, 24–27) or 46 adults (28), respectively. Regarding the age of the studied population, there is great heterogeneity, with studies analyzing ages ranging from 17 to 63 years in adults (10, 28, 35, 38, 39, 41–44, 46–49, 51) and from 3 months-18 years in children (1, 5, 9, 12, 13, 16, 24–27, 40, 42, 45, 50, 54, 57). Similar to our study, with a mean age of 30.15 ± 5.6 and 31 ± 6.3 years old for adults (non-ADC and ADC respectively) and 7.85 ± 1.18 and 8.4 ± 1.23 years old for children (non-ADC and ADC respectively), with a narrow range to avoid biases associated with age changes. Regarding the variables of interest evaluated in relation to salivary metagenomics, studies in adults have focused on caries (10, 28, 38, 39, 42, 48, 51), periodontal disease (14, 35, 44, 46, 49) or both (41, 43, 47, 52). In contrast, in children, the studies conducted evaluated caries disease (1, 5, 9, 12, 13, 16, 24–27, 40, 42, 45, 54, 57). To determine the status of oral health or disease, different diagnostic criteria and reference indices were used in published studies. In previous research the WHO criteria were employed as the diagnostic method for caries (12, 23, 24, 45), while in other studies used ICDAS method (1, 16, 27, 51).

In relation to the association between salivary biochemical parameters (salivary flow rate, pH, TPC, and TAC) and ADC in both children and adults, our analysis did not reveal any significant correlations in either age group. These findings were somewhat unexpected, given prior evidence suggesting otherwise. For instance, Pandey et al. (58) reported higher salivary pH levels in caries-free children and observed an increase in antioxidant capacity in children with active caries, irrespective of age. Consistent with our results, however, they found no significant differences in the salivary flow rate between caries-active and caries-free individuals. In contrast, Pyati et al. (59) observed significant differences in salivary flow rate, pH, and total protein levels between children with active caries and healthy controls. Similarly, a recent systematic review focusing on paediatric populations (60) supported these findings, indicating that salivary parameters such as flow rate, pH, and buffering capacity tend to decrease in children with caries, regardless of age or sex. However, the authors reported no significant changes in total protein concentration among children aged 6–12 years, which aligns with our results, especially considering the narrow age range employed in our study to minimize age-related variability.

One possible explanation for the lack of significant associations in our data could be the multifactorial nature of dental caries, which involves complex interactions between host factors, diet, oral hygiene practices, and microbiota composition. Biochemical parameters such as pH and antioxidant capacity may fluctuate over short periods and be influenced by recent food intake or stress, making them less reliable as isolated markers of caries activity. Additionally, the narrow range of biochemical values observed in our participants, most of whom exhibited values within normal physiological limits, might have limited our ability to detect statistically significant differences. It is also possible that microbial composition and ecological shifts in the biofilm play a more critical role in caries development than the salivary biochemistry alone.

Thus, we explored the relationship between salivary microbiota composition with ADC in both age groups. In the present study, we did not observe significant correlations between ADC and the relative abundance of bacterial phyla in either children or adults. These findings suggest that shifts in the salivary microbiota related to caries may occur more prominently at lower taxonomic levels, such as genus or species, rather than at the broader phylum level. This observation aligns with previous studies indicating that caries-associated microbial changes are often subtle and localized to specific taxa rather than being characterized by global shifts in major bacterial phyla (32, 61). In relation to periodontal status, although in children no statistical correlation was found, a significant and negative relationship was found between CPI and *Bacillota* and *Actinobacteriota,* according to previous research, stating that *Fusobacteria* and *Saccharibacteria* (*TM7*) are the most abundant phyla associated with gingivitis, while *Actinobacteriota* and *Bacteroidetes* are less frequently associated with gingivitis (35).

At the genus level, several taxa showed significant positive correlations with ADC in adults, including *Corynebacterium*, *Porphyromonas*, *Tanerella*, *Catonella*, *Oribacterium*, *Mogibacterium*, *Filifactor*, *Parvimonas*, *Dialister*, or *Leptotrichia*. Many of these genera have been associated with periodontal and endodontic infections, and their presence may reflect a microbial dysbiosis that supports both caries progression and other oral pathologies (62, 63). For example, *Porphyromonas* have been implicated in pulp and periapical infections and have been increasingly associated with the progression of deep carious lesions (62–67). Similarly, *Filifactor* and *Parvimonas* are anaerobic genera linked to endodontic and periodontal diseases, supporting the notion that late-stage dentin caries may share microbial signatures with these conditions. These results suggest that the adult oral microbiota harbours a more diverse array of genera associated with caries, many of which have been previously implicated in dysbiotic biofilms and periodontal or endodontic infections. The presence of multiple anaerobic and proteolytic taxa (e.g., *Filifactor*, *Mogibacterium*) in this group may reflect the complex microbial consortia contributing to caries progression beyond classical acidogenic bacteria. However, it should be noted that in our study, adults with higher ADC level also presented higher level of CPI. Thus, these findings should be confirmed in later studies. Conversely, *Haemophilus* showed a significant negative correlation with ADC in adults, and thus, its abundance could potentially protect from caries development. This genus is commonly associated with health-associated oral microbiota and may exert protective effects via competitive exclusion or production of neutral or alkali metabolic byproducts.

At the species level, in adult saliva, the presence of ADC was positively correlated with *P. gingivalis, P. endodontalis*, *P. stomatis*, *L. buccalis*, *P. oris*, and *C. matruchotii*. Interestingly, *P. gingivalis*, traditionally linked to periodontitis, and *P. stomatis*, typically associated with endodontic infections, showed positive correlations with caries in adults, suggesting potential ecological shifts in the salivary microbiome under cariogenic conditions. *C. matruchotii*, though traditionally considered a commensal, may contribute structurally to the development of cariogenic biofilms through its characteristic filamentous morphology and central positioning in the “hedgehog” structure of supragingival plaque (68). A positive correlation between *P. gingivalis* and CPI was found, according tio Kim et al. (44), who reported an increase in the group of patients with periodontitis, along with abundance of *T. denticola* and *T. forsythia*. In children, by contrast, the associations between microbiota and caries were more limited. Only the genera *Scardovia*, particularly known for its role in early childhood caries and acidogenic potential, was significantly and positively correlated with ADC. *Scardovia wiggsiae*, in particular, has been well documented as a caries-associated species in paediatric populations and has been proposed as an early colonizer in the development of severe early childhood caries (69). At the species level, *P. stomatis* was significantly and negatively associated with caries, suggesting a potentially protective role in the paediatric population. Interestingly, *P. stomatis* showed opposing correlations with caries in adults and children, positive in the former and negative in the latter. This discrepancy may reflect age-related differences in the oral microbiome's composition, maturity, and ecological dynamics. In adults, *P. stomatis* may participate in dysbiotic consortia involving anaerobic and proteolytic taxa that contribute to caries progression. In contrast, in children whose microbiome is less mature and more dominated by acidogenic species, *P. stomatis* may play a less pathogenic or even modulatory role. These divergent associations may also be influenced by differences in salivary parameters, immune responses, or microbial interactions across age groups. Further studies are needed to clarify the functional role of *P. stomatis* in distinct developmental contexts.

This study presents several limitations that should be considered when interpreting the results. First, the sequencing of the V3–V4 regions of the 16S rRNA gene, although widely used in microbiome studies, does not always provide sufficient resolution to reliably distinguish between closely related bacterial species. As a result, a substantial proportion of sequences could not be taxonomically classified at the species level, potentially limiting the precision of our microbial profiling. Second, the classification of participants was based solely on the presence or absence of ADC. While none of the individuals in the non-ADC group presented clinical signs of periodontitis, the ADC group included participants with varying periodontal health status, ranging from high periodontal indices to no clinical signs. This heterogeneity may function as a confounding factor, particularly considering the overlapping microbial profiles between caries and periodontal diseases. Future studies should aim to better control for periodontal status to isolate the specific microbial signatures associated with ADC. Third, the cross-sectional nature of this study prevents the establishment of causal relationships between microbiota composition, salivary biochemical parameters, and caries status. Longitudinal studies are needed to elucidate temporal dynamics and causality. Additionally, the sample size, although comparable to previous studies in this field, was relatively limited, which may reduce statistical power. Besides, due to the theoretical calculation of sample size the generalization could be reduced. Therefore, correlation analyses were employed as an exploratory approach to detect potential associations, recognizing the inherent limitations in generalizability and robustness. The statistical analysis of beta diversity lacks model adjusting of covariables as sex or age, due to the low sample size. Although that adjusted analysis would improve the quality of the interpretation and generability in a wide sample size, in our sample the sample size divided by sex and age would rise the biases (i.e., low data precision, atypical data, high confidence intervals, data distortion) of the data analysis. Besides, sampling was paired by sex in order to prevent sex influence in results. Furthermore, some aspects of the CPI index constitute limitations, as it is a population-based epidemiological index, but it is an index that measures the need for treatment associated with pathology. According to the WHO and the American Society of Pediatric Dentistry, its use is recommended for assessments in both adults and child populations. Besides, it has been used in previous research (30, 49, 70, 71).

Despite these limitations, this study has several notable strengths. Most existing salivary metagenomic studies have been conducted in Asian populations, where cultural practices, dietary habits, and oral hygiene behaviours differ significantly from those in other regions. Our study contributes novel data from a Western European population, specifically from Spain, where only limited microbiome data is currently available. This adds valuable intercultural insight into the salivary microbiota and its association with oral health. Moreover, the study was carefully designed to minimize confounding variables. Thus, adults and children, with and without ADC were balanced for sex and body mass index (BMI), and in both age groups, participants were matched by age to reduce potential age-related microbiome variability. Saliva collection followed standardized and validated protocols, ensuring consistency in sample handling and processing. Additionally, rigorous inclusion and exclusion criteria were applied, helping to control for variables such as recent antibiotic use, systemic diseases, and other factors known to influence the oral microbiome. Finally, the integration of microbiota data with salivary biochemical markers, such as TAC and total TPC, adds depth to the findings and provides a more holistic view of the oral ecosystem. The balanced study design, along with the novelty of exploring both microbiota and oxidative markers in relation to caries in adults and children, enhances the relevance and scientific value of this work.

Finally, interpreting the data obtained, significant differences were found in salivary bacterial composition in the presence of ADC, as well as periodontal disease, the most common diseases in dentistry. Furthermore, data suggest that salivary parameters do not play as important a role as salivary microbiological composition. However, the aforementioned limitations do not allow generalization to broad populations, making it necessary to conduct broad-spectrum, multicentre, and controlled studies that allow for firm conclusions and allow for extrapolation of data.

## 5 Conclusions

This study explored the relationship between salivary microbiota composition, salivary biochemical parameters, and ADC in both children and adults. Although no significant associations were observed between phylum-level microbial composition and ADC status, specific genera and species showed positive correlations with caries activity, particularly in adults. These included *Corynebacterium*, *Porphyromonas*, *Filifactor, Olsenella, Dialister* or *Catonella*, among other microorganisms previously associated with oral dysbiosis and disease. In children, the genus *Scardovia* was positively related to ADC. This supports the idea that distinct microbial profiles may underlie caries development in different age groups, which may be influenced by factors such as diet, oral hygiene habits, maturity of the oral microbiome, and host immune responses. In this sense, the specie *P. stomatis* showed opposing correlations with caries in adults and children, positive in the former and negative in the latter.

Despite initial hypotheses, salivary biochemical parameters, including flow rate, pH, total protein concentration, and total antioxidant capacity, did not show significant associations with ADC presence in either age group.

Taken together, these findings suggest that microbial factors may play a more significant role than salivary biochemical markers in distinguishing caries activity and suggest age-specific microbial risk markers, particularly in adults. Future studies with larger sample sizes and longitudinal designs are needed to confirm these associations and explore potential mechanistic pathways.

## Data Availability

The raw data supporting the conclusions of this article will be made available by the authors, without undue reservation.
