# Supplementary figures and images for: Salivary microbiota profile in adult and children population according to active dentin caries: a metagenomic preliminary analysis
Source: Front Oral Health. 2025 Jul 28;6:1599925. doi: 10.3389/froh.2025.1599925 (PMC12336124; doi:10.3389/froh.2025.1599925)

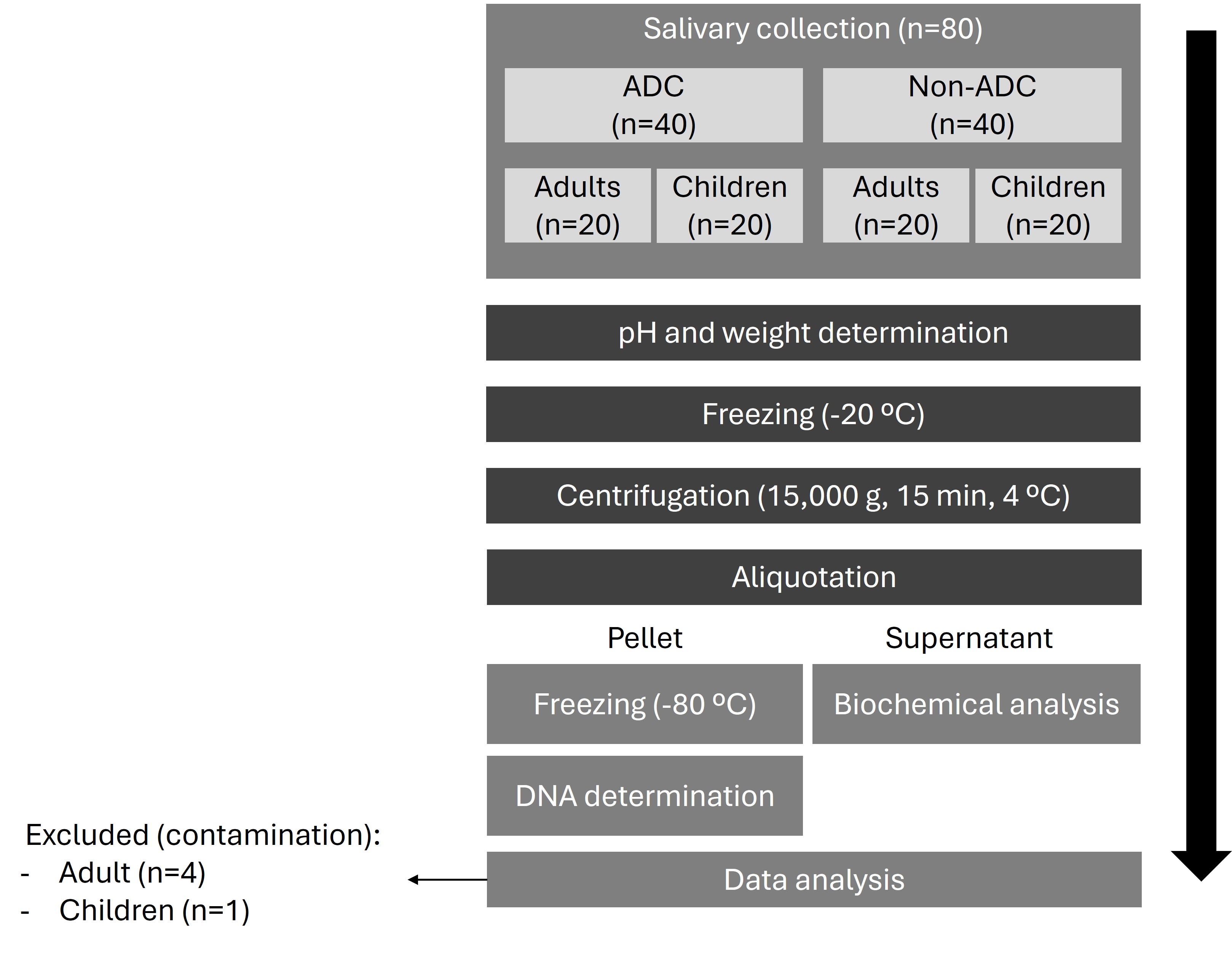

Supplement: Supplementary file 1 [file Image1.jpeg]
