# Supplementary material for: Salivary microbiota profile in adult and children population according to active dentin caries: a metagenomic preliminary analysis
Source: Front Oral Health. 2025 Jul 28;6:1599925. doi: 10.3389/froh.2025.1599925 (PMC12336124; doi:10.3389/froh.2025.1599925)

**Supplementary table S1.** STROBE Statement—checklist of items that should be included in reports of observational studies.

|  | Item No. | Recommendation | | | | Page No. | Relevant text from manuscript |
| --- | --- | --- | --- | --- | --- | --- | --- |
| **Title and abstract** | 1 | (*a*) Indicate the study’s design with a commonly used term in the title or the abstract | | | | 1 | Title |
|  |  | (*b*) Provide in the abstract an informative and balanced summary of what was done and what was found | | | | 1-2 | Abstract |
| Introduction | | | | | | |  |
| Background/rationale | 2 | Explain the scientific background and rationale for the investigation being reported | | | | 2-3 | Section 1 |
| Objectives | 3 | State specific objectives, including any prespecified hypotheses | | | | 3 | Aim |
| Methods | | | | | | |  |
| Study design | 4 | Present key elements of study design early in the paper | | | | 3 | Section 2 - beginning |
| Setting | 5 | Describe the setting, locations, and relevant dates, including periods of recruitment, exposure, follow-up, and data collection | | | | 3-5 | Sections 2.2, 2.3 and 2.4. |
| Participants | 6 | Give the eligibility criteria, and the sources and methods of selection of participants | | | | 3-4 | Section 2.1 |
| Variables | 7 | Clearly define all outcomes, exposures, predictors, potential confounders, and effect modifiers. Give diagnostic criteria, if applicable | | | | 3-4 | Sections 2.2, 2.3 and 2.4. |
| Data sources/ measurement | 8* | For each variable of interest, give sources of data and details of methods of assessment (measurement). Describe comparability of assessment methods if there is more than one group | | | | 4-5 | Sections 2.2, 2.3 and 2.4. |
| Bias | 9 | Describe any efforts to address potential sources of bias | | | | 3 | Section 2.1 |
| Study size | 10 | Explain how the study size was arrived at | | | | 4 | Section 2.1 |
| Quantitative variables | 11 | Explain how quantitative variables were handled in the analyses. If applicable, describe which groupings were chosen and why | | | | 5 | Section 2.5 |
| Statistical methods | 12 | (*a*) Describe all statistical methods, including those used to control for confounding | | | | 5 | Section 2.5 |
|  |  | (*b*) Describe any methods used to examine subgroups and interactions | | | | 5 | Section 2.5 |
|  |  | (*c*) Explain how missing data were addressed | | | | 5 | Section 2.5 |
|  |  | (*d*) *Cross-sectional study*—If applicable, describe analytical methods taking account of sampling strategy | | | | 5 | Section 2.5 |
|  |  | (*e*) Describe any sensitivity analyses | | | | - | - |
| Results |  |  | | | |  |  |
| Participants | 13* | (a) Report numbers of individuals at each stage of study—eg numbers potentially eligible, examined for eligibility, confirmed eligible, included in the study, completing follow-up, and analysed | | | | 5 | Section 3.1 |
|  |  | (b) Give reasons for non-participation at each stage | | | | 5 | Section 3.1 |
|  |  | (c) Consider use of a flow diagram | | | | 5 | Figure 1 |
| Descriptive data | 14* | (a) Give characteristics of study participants (eg demographic, clinical, social) and information on exposures and potential confounders | | | | 5 | Section 3.1 |
|  |  | (b) Indicate number of participants with missing data for each variable of interest | | | | 5 | Section 3.1 |
| Outcome data | 15* | *Cross-sectional study—*Report numbers of outcome events or summary measures | | | | *Yes* |  |
| Main results | 16 | (*a*) Give unadjusted estimates and, if applicable, confounder-adjusted estimates and their precision (eg, 95% confidence interval). Make clear which confounders were adjusted for and why they were included | | | | 5-7 | Section 3 |
|  |  | (*b*) Report category boundaries when continuous variables were categorized | | | | - | - |
|  |  | (*c*) If relevant, consider translating estimates of relative risk into absolute risk for a meaningful time period | | | | - | - |
| Other analyses | 17 | Report other analyses done—eg analyses of subgroups and interactions, and sensitivity analyses | | | | 5-7 | Section 3 |
| Discussion |  |  | |  |  |  |  |
| Key results | 18 | Summarise key results with reference to study objectives | | | | 7-11 | Section 4 |
| Limitations | 19 | Discuss limitations of the study, taking into account sources of potential bias or imprecision. Discuss both direction and magnitude of any potential bias | | | | 10-11 | Section 4 |
| Interpretation | 20 | Give a cautious overall interpretation of results considering objectives, limitations, multiplicity of analyses, results from similar studies, and other relevant evidence | | | | 11 | Section 4 |
| Generalisability | 21 | Discuss the generalisability (external validity) of the study results | | | | 11 | Section 4 |
| Other information |  |  |  | |  |  |  |
| Funding | 22 | Give the source of funding and the role of the funders for the present study and, if applicable, for the original study on which the present article is based | | | | 12 | Funding section |

**Supplementary table S2.** Descriptive statistics of the biochemistry analysis of the salivary samples.

|  | ADULTS | | | CHILDREN | | |
| --- | --- | --- | --- | --- | --- | --- |
|  | **ADC** | **Non-ADC** | ***p* value*** | **ADC** | **Non-ADC** | ***p* value** |
| pH | 6.93±0.43 | 7.03±0.18 | 0.375 | 7.15±0.35 | 7.03±0.31 | 0.276 |
| Flow rate  (ml/min) | 0.56±0.30 | 0.54±0.21 | 0.843 | 0.62±0.40 | 0.63±0.40 | 0.943 |
| TPC  (μg/μL BSA) | 1282.02±553.26 | 1318.35±348.44 | 0.805 | 1257.56±489.87 | 1055.13±303.97 | 0.224 |
| TAC  (μM FeSO4) | 349.87±109.44 | 384.75±163.33 | 0.432 | 308.26±156.79 | 255.09±111.11 | 0.125 |

SD: standard deviation. TPC: Total Protein Content. TAC: Total Antioxidant Capacity.

** p* value of Student T test, Significative values p<0.05.

**Supplementary table S3**. Alpha diversity indices between oral health status groups (ADC/non-ADC) in adults and children participants.

|  | ADULTS | | | CHILDREN | | |
| --- | --- | --- | --- | --- | --- | --- |
|  | **ADC** | **Non-ADC** | ***p* value^+^** | **ADC** | **Non-ADC** | ***p* value^+^** |
| Chao 1 | 688.47±418.28 | 722.46±531.50 | 0.66 | 454.63±258.15 | 431.55±294.64 | 0.34 |
| Observed features | 663.75±400.35 | 690.35±508.89 | 0.57 | 436.88±241.30 | 420.3±280.08 | 0.45 |
| Shannon | 6.30±0.96 | 6.07±1.10 | 0.35 | 5.99±0.43 | 5.93±0.62 | 0.51 |
| Simpson | 0.95±0.03 | 0.94±0.04 | 0.24 | 0.95±0.01 | 0.95±0.02 | 0.52 |

SD: standard deviation.  ^+^*p* value of Mann-Whitney test, significative values p<0.05.

**Supplementary table S4**. Beta diversity analysis between oral health status groups (ADC/non-ADC) in adults and children participants with PERMANOVA method.

| Group | Degree of freedom | Sums of squares | Sums of squares/degree of freedom | F-test value | R2 | P value |
| --- | --- | --- | --- | --- | --- | --- |
| Adults | 1(35) | 0.2387(6.72662) | 0.2387(0.19219) | 1.242 | 0.03427(0.96573) | 0.168 |
| Children | 1(37) | 0.0951(5.65855) | 0.0951(0.15293) | 0.6218 | 0.01653(0.98347) | 0.962 |

R2, the explanation of grouping factor on the difference of samples, calculated from the ratio of grouping variance and total variance. Values in parentheses stand for Residual Error.

**Supplementary table S5.** Spearman correlation between active dentin caries and microbial phyla with a relative abundance greater than 0.1% in both children and adults.

|  | **ADULTS** | | **CHILDREN** | |
| --- | --- | --- | --- | --- |
|  | r | p value | r | p value |
| ***Firmicutes*** | 0.121 | 0.476 | -0.075 | 0.651 |
| ***Proteobacteria*** | -0.264 | 0.114 | -0.028 | 0.864 |
| ***Bacteroidota*** | 0.213 | 0.204 | -0.068 | 0.682 |
| ***Actinobacteriota*** | -0.068 | 0.690 | 0.230 | 0.158 |
| ***Fusobacteriota*** | 0.296 | 0.076 | 0.147 | 0.369 |
| ***Campilobacterota*** | 0.114 | 0.501 | 0.110 | 0.504 |
| ***Patescibacteria*** | 0.119 | 0.480 | -0.006 | 0.972 |

**Supplementary table S6.** Spearman correlation between CPI and microbial phyla with a relative abundance greater than 0.1% in both children and adults.

|  | **ADULTS** | | **CHILDREN** | |
| --- | --- | --- | --- | --- |
|  | r | p value | r | p value |
| ***Firmicutes*** | 0.347 | 0.035* | -0.060 | 0.716 |
| ***Proteobacteria*** | -0.234 | 0.163 | 0.075 | 0.650 |
| ***Bacteroidota*** | 0.218 | 0.194 | 0.045 | 0.785 |
| ***Actinobacteriota*** | -0.335 | 0.043* | 0.015 | 0.928 |
| ***Fusobacteriota*** | 0.268 | 0.109 | -0.128 | 0.439 |
| ***Campilobacterota*** | 0.068 | 0.691 | 0.083 | 0.617 |
| ***Patescibacteria*** | 0.090 | 0.598 | 0.053 | 0.751 |

** p* value of Spearman correlation test, Significative values p<0.05.

**Supplementary figure F1.** Flow chart of sampling procedure.


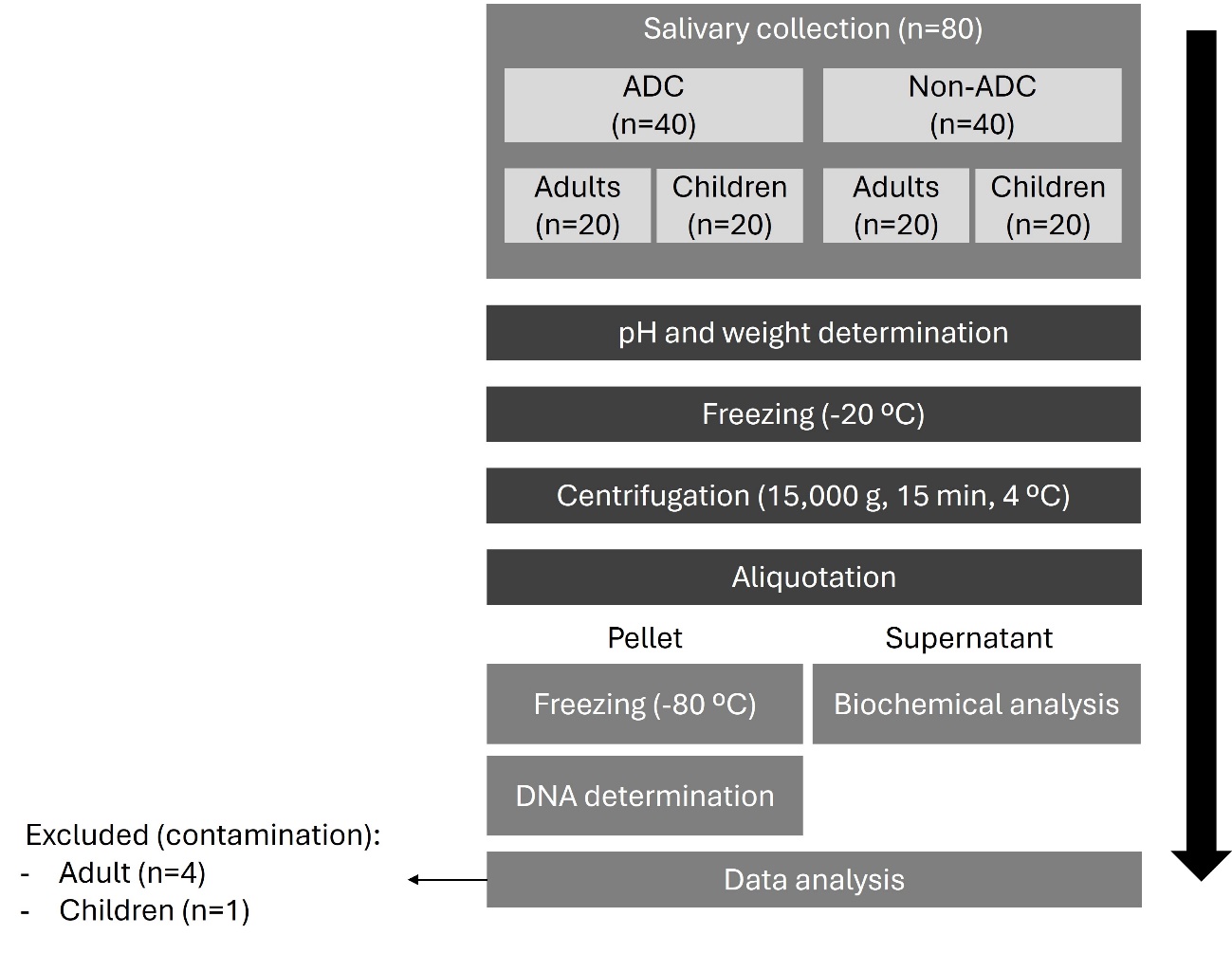

Supplement: Supplementary file 2 [file Table1.docx]
